# Vitamin D nutrition of healthy schoolchildren from North India

**DOI:** 10.1186/1687-9856-2015-S1-P65

**Published:** 2015-04-28

**Authors:** Kriti Joshi, Vijayalakshmi Bhatia, Nirupama Singh, V Ramesh

**Affiliations:** 1 Department of Endocrinology, Sanjay Gandhi Post Graduate Institute of Medical Sciences, Lucknow, India; 2 Department of Dietetics, SGPGIMS, Lucknow, India; 3 Department of Pathology, SGPGIMS, Lucknow, India

## 

Vitamin D deficiency is not expected in prepubertal schoolgoing children in a sunny country like India since they should have good sunshine exposure in conjunction with school activities. However data from India in this age group are scant.

## Aims

To evaluate the vitamin D status of apparently healthy prepubertal vs pubertal schoolchildren and to examine the influence of gender and socioeconomic status on serum 25 hydroxyvitamin D (25OHD).

## Methods

Children referred to the pediatric endocrinology clinic for growth evaluation, interpretation of thyroid functions, concern regarding delayed puberty, etc and found to be normal were enrolled, as were their healthy siblings. Pubertal assessment, a food frequency questionnaire for calcium, sunlight exposures (minutes of sunshine exposure from 10 AM - 4PM) and percentage of skin exposed were recorded. Serum calcium, albumin, creatinine, alkaline phosphatase (ALP), 25OHD and iPTH were assayed.

## Results

118 children (69% prepubertal ) were enrolled. Vitamin D deficiency (25OHD < 20 ng/ml) was present in 71.6% of prepubertal and 87.8% of pubertal children. Mean 25 OHD was significantly higher in the prepubertal as compared to the pubertal group (16.0 ± 7.9 versus 10.6 ± 7.5 ng/ml, p = 0.001). Their calcium intake and sunlight exposure was also significantly higher. Girls had lower mean 25 OHD as compared with boys (12.9 ± 7.6 vs 16.0 ± 8.4, p=0.02), lower calcium intake (569 ± 270 vs 712 ± 334 mg, p=0.02) and less sunlight exposure (46.7 ± 45.8 vs 75.1 ± 39.1 minutes, p=0.001) than their male counterparts. There was no significant difference in 25OHD of upper, middle and lower socioeconomic classes. We found a significant correlation between serum 25OHD and calcium intake (r: 0.238, p=0.02), duration of sunlight exposure (r: 0.235, p=0.02) and body surface area exposed (r: 0.28, p=0.003). There was a significant negative correlation between 25OHD and PTH levels (r: -0.45, p<0.001)

## Conclusions

Pubertal children have lower 25OHD than prepubertal children, probably due to limitation of outdoor activities on the background of high demand. There is significant gender bias against girls for both calcium intake and sun exposure, resulting in lower serum 25OHD than in boys.

